# Effects of Photosynthesis Inhibitors on H_2_ Production in Microalgae and Cyanobacteria

**DOI:** 10.3390/plants15132012

**Published:** 2026-06-29

**Authors:** Dariga K. Kirbayeva, Assemgul K. Sadvakasova, Dauren Botbayev, Meruyert O. Bauenova, Dilnaz E. Zaletova, Ayaz M. Belkozhayev, Altynbek S. Abseyt, Fiaz Ahmad, Bekzhan D. Kossalbayev

**Affiliations:** 1 Department of Biotechnology, Faculty of Biology and Biotechnology, Al-Farabi Kazakh National University, Al-Farabi Ave. 71/19, Almaty 050040, Kazakhstan; kk.dariga@gmail.com (D.K.K.); asem182010@gmail.com (A.K.S.); bauyen.meruyert@gmail.com (M.O.B.); zaletova_dilnaz@mail.ru (D.E.Z.); 2 Department of Chemical and Biochemical Engineering, Geology and Oil-Gas Business Institute Named After K. Turyssov, Satbayev University, Almaty 050043, Kazakhstan; a.belkozhayev@satbayev.university; 3 M.A. Aitkhozhin Institute of Molecular Biology and Biochemistry, Almaty 050012, Kazakhstan; 4 Ecology Research Institute, Khoja Akhmet Yassawi International Kazakh-Turkish University, Turkistan 161200, Kazakhstan; altynbek.abseyt@bk.ru; 5 Key Laboratory for Space Bioscience & Biotechnology, School of Life Science and Technology, Northwestern Polytechnical University, Chang’an Campus, Xi’an 710072, China; fiaz.a@nwpu.edu.cn

**Keywords:** photobiological H_2_ production, microalgae, cyanobacteria, photosynthesis inhibitors, DCMU, DBMIB, CCCP, atrazine

## Abstract

Photobiological hydrogen (H_2_) production by microalgae and cyanobacteria is widely seen as a promising and sustainable alternative to H_2_ produced from fossil fuels. However, its efficiency remains limited because the enzymes responsible for H_2_ production are highly sensitive to oxygen (O_2_), while photosynthesis itself generates O_2_ that can suppress their activity. This mini-review explores how different photosynthesis inhibitors affect H_2_ production in these microorganisms, with a focus on their molecular targets and their physiological effects. In both microalgae and cyanobacteria, compounds such as DCMU, atrazine, DBMIB, CCCP, and KCN influence H_2_ metabolism by altering electron transport, O_2_ release, proton gradients, and cellular redox balance. The reviewed studies indicate that complete inhibition of photosynthetic electron flow is usually unfavorable for sustained H_2_ production because it reduces the electron supply required by H_2_-evolving enzymes. Therefore, anaerobiosis is more reliably established by physiological or cultivation-based strategies, whereas photosynthetic and respiratory inhibitors are best used as mechanistic probes or as auxiliary modulators under carefully optimized conditions. Their effects are strongly context-dependent, reflecting the balance between O_2_ suppression, residual electron transport, respiratory O_2_ consumption, and competing electron sinks.

## 1. Introduction

Microalgae and cyanobacteria are promising platforms for clean energy production due to their rapid growth and efficient carbon dioxide (CO_2_) fixation [1,2]. These photosynthetic organisms convert solar energy into biomass and energy-rich compounds. Notably, the green alga *Chlamydomonas reinhardtii* (*C. reinhardtii*) possesses highly active [FeFe]-hydrogenases (H_2_ases) (HydA1, HydA2) and a photosynthetic apparatus capable of utilizing a broad light spectrum from UV to far-red wavelengths [2,3,4]. Harnessing sunlight to produce H_2_ from water is particularly attractive, as H_2_ has a high energy yield per unit mass and produces no CO_2_ upon utilization [5,6,7]. Photobiological H_2_ production using microalgae and cyanobacteria offers a promising solution to this challenge. Cyanobacteria, like microalgae, can produce H_2_ through the activity of H_2_ase and nitrogenase (N_2_ase) enzymes. In these prokaryotic phototrophs, H_2_ metabolism is closely linked to photosynthetic electron transport and O_2_ evolution [8]. As a result, both microalgae and cyanobacteria serve as important model systems for studying and improving biological H_2_ production. In photosynthetic microorganisms, H_2_ is generated when electrons derived from water splitting at photosystem II (PSII) are transferred via ferredoxin to H_2_ase [9]. However, PSII simultaneously produces molecular O_2_ (O_2_), which irreversibly inactivates O_2_-sensitive H_2_ases [10]. Consequently, efficient H_2_ production typically requires anaerobic or nutrient-stress conditions that suppress PSII activity and reduce intracellular O_2_ levels [11]. This creates a fundamental challenge: photosynthesis provides the electrons necessary for H_2_ production, while at the same time generating O_2_ that inhibits H_2_ase activity. Overcoming this contradiction is therefore a key scientific problem in photobiological H_2_ research [12,13]. One experimental strategy to investigate this limitation is the use of photosynthetic and respiratory inhibitors that perturb defined steps of electron transport. However, these compounds should not be regarded as universal inducers of anaerobic H_2_-producing conditions, because inhibition of photosynthetic electron transport can simultaneously suppress O_2_ evolution and restrict the electron supply required by H_2_ase or N_2_ase. Therefore, inhibitor studies are most informative when they are used to dissect electron-transfer routes under defined hypoxic, anaerobic, or H_2_-producing conditions.

Inhibitors of photosynthesis should be interpreted as mechanistic probes rather than universal enhancers of H_2_ production. For each inhibitor, the biologically relevant sequence is: molecular target and binding site, immediate effect on electron transfer and O_2_ evolution, redox consequences for the PQ/PQH_2_ pool and stromal acceptor side, and resulting change in electron availability for hydrogenase or nitrogenase. This distinction is essential because the same treatment may reduce O_2_ inhibition while simultaneously restricting the photosynthetic reductant required for H_2_ evolution [14].

Classical inhibitors such as 3-(3,4-dichlorophenyl)-1,1-dimethylurea (DCMU), which blocks electron transfer at the PSII acceptor site, and 2,5-dibromo-3-methyl-6-isopropyl-*p*-benzoquinone (DBMIB), which inhibits electron transport at the plastoquinone cytochrome b_6_f complex, are widely used for this purpose [15,16]. Other inhibitors targeting PSII include atrazine, which competes with plastoquinone at the QB binding site and suppresses O_2_ evolution [17]. In addition, protonophores such as carbonyl cyanide *m*-chlorophenyl hydrazone (CCCP) disrupt the proton gradient across the thylakoid membrane, thereby uncoupling electron transport from ATP synthesis and indirectly affecting H_2_ metabolism [18]. Furthermore, compounds acting at different levels of cellular metabolism have also been reported. For example, photosystem I (PSI) electron acceptors such as paraquat can redirect electrons from PSI, leading to reactive O_2_ species formation and altered redox balance. Similarly, respiratory inhibitors such as potassium cyanide have been used, particularly in cyanobacteria, to suppress competing electron sinks and influence H_2_ production pathways [19,20]. Previous studies have shown that low or delayed DCMU application can prolong H_2_ production under specific conditions, probably by reducing O_2_ evolution while residual or alternative electron sources remain available (Table 1). Nevertheless, this effect is strongly dependent on concentration, timing, physiological state, and culture conditions. Therefore, photosynthesis inhibitors are better described as tools for probing or fine-tuning electron allocation under H_2_-producing conditions, rather than as general substitutes for physiological anaerobic induction [21]. In this context, photosynthesis inhibitors can function as tools to artificially mimic stress-induced anaerobic states. As a result, chemical modulation of photosynthetic electron transport has become an important experimental strategy for enhancing H_2_ production in microalgae and cyanobacteria [22,23]. Despite significant progress in this field, the specific roles of different classes of photosynthesis inhibitors in regulating electron allocation and H_2_ase activity remain insufficiently systematized [24].

**Figure 1:**
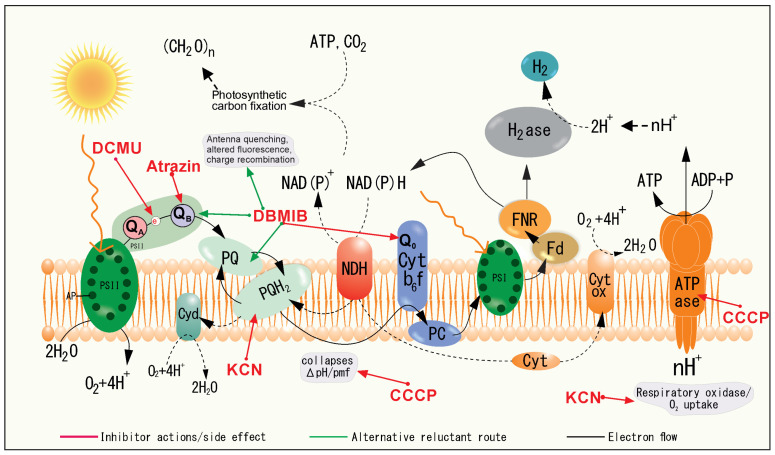
Photosynthetic electron transport chain and inhibitor action sites regulating photobiological H_2_ production. Electrons from water oxidation at PSII are transferred through Q_A_/Q_B_, the PQ/PQH_2_ pool, Cyt b_6_f, PC, PSI, and Fd to H_2_ase for H_2_ production. Fd also supplies electrons to FNR for NAD(P)H formation and carbon fixation. DCMU and atrazine inhibit the PSII Q_B_ acceptor side. DBMIB mainly inhibits PQH_2_ oxidation at the Q_o_ site of Cyt b_6_f and may additionally affect PSII fluorescence, charge recombination, and quinone-mediated reactions. CCCP dissipates ΔpH/pmf and uncouples ATP synthesis. KCN inhibits respiratory oxidases and O_2_-consuming electron sinks. Molecular O_2_ strongly inhibits H_2_ase. Black arrows indicate electron flow, red arrows indicate inhibitor actions or side effects, and green arrows indicate alternative reductant routes [25,26,27,28].

This mini-review aims to summarize current knowledge on the effects of photosynthesis inhibitors on H_2_ production in microalgae and cyanobacteria, with particular emphasis on their mechanisms of action and their impact on photosynthetic electron transport and H_2_ metabolism (Figure 1).

## 2. Effects of Photosynthesis Inhibitors in Microalgae

### 2.1. Photosystem II Inhibitors: DCMU and Atrazine

Photosynthesis inhibitors have been widely used as experimental tools to reduce O_2_ evolution and to identify electron transport routes involved in H_2_ production. However, they should not be considered universal or preferred methods for establishing anaerobiosis, because inhibition of photosynthetic electron transport can simultaneously suppress the electron supply required by H_2_ase or N_2_ase. In many systems, anaerobic or hypoxic conditions are more reproducibly achieved by physiological or cultivation-based strategies, such as sulfur deprivation, dark anaerobic incubation, inert gas flushing, high-cell-density respiration, immobilization, or engineered hypoxic microenvironments. Under these conditions, inhibitors are most useful as mechanistic probes to distinguish whether H_2_ production is limited by O_2_ evolution, electron availability, proton-gradient formation, or competing electron sinks [29,30,31].

The inhibitor DCMU binds to the Q_B_-binding site of the D1 protein in the PSII reaction center and blocks electron transfer from the primary to the secondary quinone acceptor. As a result, PSII-dependent electron transfer and O_2_ evolution are suppressed. This can reduce intracellular O_2_ tension, but it simultaneously limits the supply of PSII-derived electrons to downstream carriers and H_2_ase. Thus, DCMU is useful for testing whether H_2_ production depends on PSII-derived electrons, but its use as an inducer of anaerobiosis must be interpreted cautiously [32]. However, this also disrupts electron flow from PSII. The addition of DCMU to *C. reinhardtii* reduces photobiological H_2_ production by blocking electron transfer from water photolysis, indicating that H_2_ase activity largely depends on PSII-derived electrons. In contrast, maintaining electron transport while creating localized anaerobic conditions is a more effective strategy. For example, Li et al. (2026) demonstrated that a hydrogel–microorganism system enables efficient H_2_ production without direct PSII inhibition [33]. In studies by other authors, H_2_ production increased following a delayed, dose-dependent addition of DCMU in *Chlorella pyrenoidosa* cultures, typically by ~1.5–2-fold depending on experimental conditions [25,26]. However, these experiments were conducted using earlier protocols and under cultivation conditions without stress factors, such as sulfur deprivation. In contrast, more recent findings indicate that during prolonged cultivation, DCMU does not maintain sustained O_2_ depletion, and H_2_ production rapidly declines [34,35,36]. Under such conditions, alternative metabolic pathways, such as acetate assimilation and starch degradation, may partially compensate for electron supply and support H_2_ production. Recent studies have shown that under photoinhibitory conditions, PSII remains partially active and continues to contribute to H_2_ase activity. For example, Volgusheva et al. (2025) demonstrated that the contribution of PSII-derived electrons to H_2_ production increased over time, despite reduced photosynthetic efficiency [37]. These findings suggest that maintaining residual PSII activity, rather than complete inhibition, is important for sustaining H_2_ production in microalgae. Similarly, Xu et al. (2023) developed a cellular bionic system in which living algal cells were coated with a conductive PPy/CaCO_3_ shell, creating a localized hypoxic microenvironment that stabilizes H_2_ase activity [38]. This engineered micro-niche enabled sustained H_2_ production and allowed extracellular photoelectrons to be directly utilized by H_2_ase, resulting in H_2_ evolution even under photosynthesis-independent conditions. These findings demonstrate that engineered microenvironments can promote H_2_ production by simultaneously reducing O_2_ levels and enabling alternative electron transfer pathways, thereby decreasing dependence on photosynthetic electron flow.

In addition to DCMU, other PSII-targeting inhibitors have also been used to investigate the regulation of electron transport and O_2_ evolution in microalgae. Although DCMU and atrazine are both classified as PSII inhibitors acting at the acceptor side, they should not be considered functionally identical. DCMU primarily blocks electron transfer between Q_A_ and Q_B_ by binding to the Q_B_ niche of the D1 protein, leading to a strong suppression of linear electron flow and O_2_ evolution. In contrast, atrazine also targets the QB-binding region of the D1 protein but differs in binding affinity, inhibitory potency, and its influence on plastoquinone reduction dynamics and electron backflow reactions. As a result, the physiological and redox responses to these inhibitors may differ depending on concentration, exposure time, and species-specific characteristics [39,40].

Among these, atrazine represents a widely studied compound with a similar but distinct mode of action [41]. Atrazine is a triazine herbicide that binds to the Q_B_ site of PSII and blocks electron transfer from plastoquinone, like DCMU. In microalgae, this results in rapid loss of PSII activity, reduced photosynthetic efficiency, and accumulation of reduced intermediates [42,43]. Recent studies confirm these effects. For example, Ospina Calvo et al. (2025) exposed the green alga *Parachlorella kessleri* (*P. kessleri*) to sub-micromolar concentrations of atrazine and observed a significant decline in the maximum quantum efficiency of PSII (Fv/Fm) and the effective PSII quantum yield (ΦPSII), indicating strong inhibition of photosynthetic electron transport [44]. Similarly, Debroy et al. (2025) exposed *Chlorella* sp. to increasing atrazine concentrations and reported dose-dependent declines in photosynthetic performance, including reductions in effective PSII quantum yield (Y(II)) and electron transport rate (ETR), indicating progressive inhibition of PSII [45].

The use of DCMU in H_2_-production studies is primarily aimed at dissecting the origin of electrons supporting H_2_ evolution. By blocking electron transfer from Q_A_ to Q_B_ at PSII, DCMU prevents the delivery of PSII-derived electrons to the photosynthetic electron transport chain while simultaneously suppressing O_2_ evolution. Consequently, comparison of H_2_ production in the presence and absence of DCMU allows researchers to distinguish between PSII-dependent (direct biophotolysis) and PSII-independent pathways that utilize electrons derived from stored carbon reserves, chlororespiration, or alternative metabolic routes. If H_2_ production is strongly inhibited by DCMU, PSII-derived electrons constitute the major source of reductant for H_2_ase activity. Conversely, sustained H_2_ evolution in the presence of DCMU indicates a significant contribution of alternative electron donors and indirect pathways. Therefore, DCMU is widely used as a diagnostic tool for probing electron allocation during H_2_ production rather than as a reliable inducer of H_2_ evolution [34].

Importantly, the effect of DCMU on H_2_ production is highly dependent on experimental conditions. Low concentrations or delayed addition may promote H_2_ evolution by reducing O_2_ accumulation and facilitating anaerobiosis, whereas complete inhibition of PSII limits the supply of photosynthetic electrons required for H_2_ase activity. Consequently, studies have reported both stimulation and inhibition of H_2_ production following DCMU treatment. These contrasting responses depend on inhibitor concentration, timing of application, physiological state of the cells, availability of alternative electron donors such as starch-derived reductants, and cultivation conditions, including sulfur deprivation and light intensity [34,46].

In this review, the term “context-dependent” refers to the fact that the effect of an inhibitor on H_2_ production depends on the biological model, the H_2_-producing enzyme involved, inhibitor concentration, exposure duration, timing of addition, light regime, nutrient status, respiratory O_2_ consumption, and the availability of alternative electron donors such as starch, acetate, or reduced stromal carriers. Thus, the same inhibitor may either stimulate, prolong, or suppress H_2_ evolution depending on whether O_2_ suppression outweighs the loss of electron supply to the H_2_-producing enzyme.

### 2.2. Inhibitors of Intersystem Electron Transport in Microalgae: DBMIB 

DBMIB should be defined as a mechanistically complex redox-active quinone analogue rather than as a simple single-site inhibitor [11,47,48,49,50]. Its classical and primary target is the quinol oxidation site, Qo, of the cytochrome b_6_f complex, where it inhibits plastoquinol (PQH_2_) oxidation and thereby restricts electron transfer from the PQ/PQH_2_ pool to the Rieske iron–sulfur protein, cytochrome f, plastocyanin, and PSI. This immediately alters the redox poise of the intersystem electron transport chain: the PQ pool becomes more reduced, electron delivery to PSI and ferredoxin becomes restricted, and the supply of electrons to hydrogenase through the PSI–ferredoxin route can decrease [51]. Therefore, when DBMIB is added during an already established H_2_-producing phase, strong inhibition usually indicates that continued electron transfer through the PQ–cytochrome b_6_f–PSI segment is required to sustain hydrogenase reduction.

However, the physiological interpretation of DBMIB cannot be limited to “PQH_2_ oxidation inhibition.” As emphasized by Vilyanen et al., [26] DBMIB has multiple side effects that complicate assigning its action solely to inhibition of PQH_2_ oxidation at cytochrome b_6_f, especially in intact cells and in vivo physiological studies. These additional effects include perturbation of PSII fluorescence signals, modification of charge recombination behavior, and quinone-mediated side reactions that may affect interpretation of electron transport measurements. Ralph et al. [52] also highlighted that DBMIB can interfere with the PSII QB side, located on the oxidizing side of the PQ pool. Therefore, DBMIB should be treated as a multi-effect probe of the thylakoid redox network, not as a clean and exclusive cytochrome b_6_f inhibitor.

This mechanistic complexity explains why DBMIB has produced apparently contrasting effects on H_2_ production. At sufficiently strong inhibition or when added after anaerobic H_2_ production has been established, DBMIB can suppress H_2_ evolution by starving PSI, ferredoxin, and hydrogenase of upstream electrons. In contrast, under carefully optimized low-dose conditions, partial restriction of intersystem electron transport may lower O_2_ evolution, alter PQ redox poise, and help maintain suboxic conditions without completely disconnecting PSI from electron supply. Thus, the effect of DBMIB on H_2_ production is not simply “stimulatory” or “inhibitory”; it depends on whether partial O_2_ suppression and redox tuning outweigh the loss of electron delivery to the PSI–ferredoxin–hydrogenase pathway.

Krishna et al. [53] demonstrated this point in sulfur-deprived, H_2_-producing *C. reinhardtii* cells. In their experimental design, anaerobic H_2_ production was first induced by sulfur deprivation, and inhibitors were then added during the established H_2_-producing phase. Under these conditions, 5 μM DBMIB almost completely suppressed H_2_ production, indicating that electron transfer through the PQ pool, cytochrome b_6_f, and PSI is required for efficient electron delivery to H_2_ase. Thus, DBMIB is especially useful for testing whether the PQ-cytochrome b_6_f-PSI segment remains necessary during H_2_ production, but its effect on H_2_ yield should not be generalized as stimulatory.

Khosravitabar and Mamedov (2023) reported that low-dose DBMIB treatment (3.5 μM) can support prolonged H_2_ production in *C. reinhardtii* under carefully optimized conditions. At low micromolar concentrations, DBMIB primarily inhibits the cytochrome b_6_f complex, whereas at higher concentrations its non-specific effects, including interactions with PSII, become more pronounced. In long-term cultures, DBMIB-treated cells maintained H_2_ production for up to 30 days, with periodic bursts of H_2_ evolution (e.g., a peak of 23 mL H_2_ L^−1^ day^−1^ on day 6) superimposed on a low basal production level [54]. A follow-up study by the same group investigated the effect of DBMIB on immobilized *C. reinhardtii* cells entrapped in alginate beads. Treatment with a low concentration of DBMIB (3.5 μM) significantly increased H_2_ production, with immobilized cells reaching approximately 200 μmol H_2_ mg^−1^ *Chl* a over three weeks, corresponding to ~2-fold higher H_2_ production compared to free-cell cultures. These findings indicate that the combination of partial intersystem electron transport inhibition and cell immobilization enhances H_2_ production, likely by stabilizing photosynthetic activity and maintaining favorable redox conditions [55]. This observation is consistent with the known biochemical mechanism of DBMIB, which involves inhibition of PQH_2_ oxidation at the Q_o_ site of the cytochrome b_6_f complex, leading to over-reduction in the plastoquinone pool and restriction of electron flow to PSI [49,50].

These recent results do not contradict the inhibitory effect observed by Krishna et al. [53], rather, they indicate that DBMIB has a narrow concentration- and system-dependent window in which partial cytochrome b_6_f limitation may tune electron flow and O_2_ evolution without fully disconnecting PSI and H_2_ase from upstream electron sources. Therefore, low-dose DBMIB treatment from the beginning of cultivation should be discussed as a specific optimization strategy, whereas DBMIB addition after anaerobic H_2_ induction remains more appropriate for mechanistic pathway dissection.

### 2.3. Proton Gradient Uncouplers in Microalgae: CCCP

CCCP should be described as a protonophore uncoupler rather than as a site-specific inhibitor of photosynthetic electron transport. Its primary action is the dissipation of the proton gradient and proton motive force across the thylakoid membrane. In its protonated form, CCCP can diffuse through the lipid phase of the membrane and release protons on the opposite side; repeated proton shuttling collapses the trans-thylakoid ΔpH and weakens the proton motive force required for ATP synthesis [56,57]. Therefore, CCCP does not directly block PSII, the PQ/PQH_2_ pool, cytochrome b_6_f, PSI, or ferredoxin. Instead, it uncouples electron transport from photophosphorylation.

The collapse of ΔpH has several important consequences for photosynthetic regulation. First, ATP synthesis through ATP synthase decreases because the proton gradient is no longer efficiently converted into ATP. Second, ΔpH-dependent photosynthetic control at the cytochrome b_6_f complex is weakened. Under normal conditions, lumen acidification slows plastoquinol oxidation at cytochrome b_6_f and prevents over-reduction in downstream carriers. CCCP releases this control by dissipating lumen acidification, thereby altering the rate of electron transport, the redox state of the PQ/PQH_2_ pool, and electron pressure on PSI acceptors. Third, the ATP/NADPH ratio becomes disturbed because reducing equivalents may still be generated while ATP production is restricted. This imbalance reshapes stromal metabolism and changes the distribution of electrons among carbon fixation, respiratory/chlororespiratory pathways, and H_2_ase-dependent H_2_ evolution [14].

The stimulation of H_2_ production by CCCP should therefore be placed at this secondary metabolic level. By lowering ATP availability, CCCP can suppress ATP-demanding competing processes such as CO_2_ fixation through the Calvin–Benson cycle, nitrogen assimilation, biosynthesis, and other energy-consuming pathways. When these sinks are restricted, reducing equivalents may become more available for alternative electron sinks, including hydrogenase. At the same time, CCCP-induced uncoupling can accelerate the transition toward low-O_2_ conditions by disturbing photosynthetic energy balance and modifying O_2_ evolution and respiratory O_2_ consumption. Under suitable hypoxic or anaerobic conditions, these combined effects can favor activation of O_2_-sensitive H_2_ase and redirect part of the electron flux toward H_2_ production [58,59].

However, this effect should not be interpreted as direct or universally beneficial stimulation. CCCP does not specifically activate H_2_ase and does not provide a dedicated electron route to H_2_ production. Its positive effect depends on whether redox redistribution and O_2_ depletion outweigh the negative consequences of ATP limitation, impaired metabolic maintenance, and possible photophysiological stress. At excessive concentrations or prolonged exposure, CCCP may suppress H_2_ evolution because severe ATP depletion can impair cell viability, photosynthetic repair, carbon metabolism, and sustained electron supply. Thus, CCCP-induced H_2_ production is best interpreted as a context-dependent systems-level response caused by proton-gradient collapse, energy uncoupling, redox redistribution, reduced competition for electrons, and altered O_2_ balance rather than inhibition of a single electron-transfer step.

Experimental studies have demonstrated that CCCP can effectively induce H_2_ production in microalgae. Yang et al. (2014) showed that the addition of 15 μM CCCP to *C. reinhardtii* cultures resulted in a 13-fold increase in short-term H_2_ production compared to untreated controls, accompanied by a complete inhibition of PSII photochemical activity [59]. Metabolomic analysis further revealed extensive metabolic reprogramming under CCCP treatment, including depletion of amino acids and unsaturated fatty acids, consistent with the establishment of anoxic conditions. Further evidence indicates that CCCP acts synergistically with other stress-based strategies that promote H_2_ production. For example, CCCP has been shown to enhance H_2_ evolution when combined with sulfur deprivation or dark–light transition regimes, where rapid dissipation of the proton gradient accelerates the onset of anaerobic conditions and redirects electron flow toward H_2_ase [60,61,62]. Similarly, in *Platymonas subcordiformis* (*P. subcordiformis*), a two-stage protocol involving dark anaerobic incubation followed by illumination, particularly under sulfur deprivation, resulted in a significant enhancement of H_2_ evolution (up to 13-fold), highlighting the critical role of anaerobic induction and sulfur limitation in activating H_2_ase activity and sustaining H_2_ production [63]. In addition, CCCP-induced uncoupling in *P. subcordiformis* was shown to rapidly inhibit PSII activity and suppress O_2_ evolution, thereby promoting intracellular anaerobiosis and protecting O_2_-sensitive H_2_ase, while simultaneously enhancing electron availability from water photolysis for H_2_ production [64]. In such systems, CCCP suppresses ATP-dependent metabolic processes and facilitates intracellular O_2_ depletion, thereby stabilizing H_2_ase activity and prolonging H_2_ production. CCCP is therefore useful as a chemical tool for testing how the proton motive force, ATP limitation, redox balance, and O_2_ depletion influence H_2_ production. Its value lies mainly in mechanistic dissection and short-term modulation, rather than as a broadly applicable strategy for sustained H_2_ production.

### 2.4. Alternative Electron Transfer Pathways and Competing Electron Sinks in Microalgal H_2_ Production

H_2_ production in eukaryotic microalgae is regulated not only by the inhibition of O_2_ evolution, but also by the distribution of photosynthetic electrons among several competing pathways. In *C. reinhardtii*, electrons generated by linear photosynthetic electron transport can be transferred from PSII through the plastoquinone pool, cytochrome b_6_f complex, PSI, and ferredoxin to [FeFe]-H_2_ase, resulting in H_2_ evolution. However, the same electron carriers also supply alternative metabolic and photosynthetic processes, including ferredoxin–NADP^+^ reductase-dependent NADPH formation, the Calvin–Benson–Bassham cycle, cyclic electron flow around PSI, chlororespiration, starch metabolism, and mitochondrial electron sinks. Therefore, the efficiency of H_2_ production depends on the balance between electron delivery to H_2_ase and electron consumption by competing pathways.

The Calvin–Benson–Bassham cycle represents one of the major stromal competitors for reducing equivalents. Under normal photosynthetic conditions, electrons from PSI are used to reduce NADP^+^ to NADPH, which is then consumed for CO_2_ fixation. When carbon fixation is active, a substantial fraction of photosynthetic reductant is directed toward biomass formation rather than H_2_ase. Conversely, under sulfur deprivation, nutrient limitation, anaerobiosis, or carbon-limited conditions, suppression of CO_2_ fixation and changes in carbon metabolism can redirect part of the electron flux toward H_2_ase. Recent work has shown that alternative photosynthetic electron pathways, including cyclic, pseudo-cyclic, and chloroplast-to-mitochondrion electron flows, are essential for maintaining ATP/NADPH balance and sustaining CO_2_ fixation in *C. reinhardtii*. This indicates that pathways supporting carbon fixation may indirectly compete with H_2_ production by consuming energy and reducing equivalents that could otherwise be available for H_2_ase activity [65].

Cyclic electron flow around PSI is particularly important in this context. In *C. reinhardtii*, PGR5/PGRL1-dependent cyclic electron flow transfers electrons from stromal carriers back to the plastoquinone pool and cytochrome b_6_f complex, thereby increasing proton translocation and ATP synthesis without producing NADPH. Although this pathway protects photosynthesis and supports energy balance, it can also restrict H_2_ production by increasing the transthylakoid proton gradient and limiting electron flow through cytochrome b_6_f toward PSI and ferredoxin. Tolleter et al. demonstrated that the proton gradient generated by cyclic electron flow strongly limits electron supply to H_2_ase, while disruption of PGRL1 releases this limitation and enhances H_2_ photoproduction. Thus, CEF should be considered a key competing and regulatory pathway rather than a secondary side process [66].

Genetic studies further support the importance of this regulatory layer. Deletion of PGR5 and PGRL1 has been shown to promote sustainable light-driven H_2_ production under sulfur deprivation. In these mutants, increased H_2_ production was associated with faster establishment of anaerobiosis, increased O_2_ consumption capacity, and improved stability of PSII activity under H_2_-producing conditions. Steinbeck et al. concluded that the enhanced H_2_ production in *pgr5* and *pgrl1* mutants results from increased PSII stability, which permits more sustained electron supply to H_2_ase. However, the effect is not simply due to CEF blockage alone; it also reflects broader changes in respiration, O_2_ consumption, photosystem stability, and redox poise [67].

Recent studies further emphasize the biotechnological relevance of PGR5-dependent regulation. Thin-cell-layer cultures of *C. reinhardtii* pgr5 and pgrl1 mutants showed enhanced H_2_ production under high-light or sunlight-intensity conditions. In addition, the pgr5 mutant was recently reported to sustain photoautotrophic H_2_ production under simulated daily light conditions, indicating that modification of alternative electron transport can improve H_2_ evolution under more realistic illumination regimes. These findings show that regulation of cyclic electron flow is a promising strategy for improving algal H_2_ production, especially when combined with controlled light exposure and optimized redox conditions [68].

Pharmacological evidence is also consistent with this interpretation. Antimycin A, which affects antimycin-sensitive cyclic electron flow, has been reported to stimulate H_2_ production under sulfur-deprived conditions by restricting electron recycling around PSI and favoring electron allocation toward H_2_ase. However, the effect of antimycin A can be strain- and condition-dependent because its impact on cyclic electron flow differs among *C. reinhardtii* strains. Therefore, antimycin A should be discussed not as a universal H_2_ enhancer, but as evidence that CEF can compete with H_2_ase-dependent electron use under specific physiological conditions [69,70].

In contrast to CEF, the chloroplastic type-II NAD(P)H dehydrogenase pathway can support H_2_ production under certain conditions. In *C. reinhardtii*, NDA2/NDH-2 reduces the plastoquinone pool using stromal NAD(P)H, especially during sulfur deprivation or when starch-derived reductants are available. This pathway contributes to PSII-independent, indirect H_2_ production because electrons stored previously in carbohydrates can be reintroduced into the photosynthetic electron transport chain through the plastoquinone pool and then transferred to PSI, ferredoxin, and H_2_ase. RNA-interference lines with lower NDA2 expression showed reduced H_2_ production, while plastidial overexpression of NDA2 increased nonphotochemical plastoquinone reduction and enhanced the rate of H_2_ production by the indirect pathway when stromal electron donors were not limiting. Thus, unlike CEF, NDA2/NDH-2 can function as an electron-supplying pathway for H_2_ production rather than only as a competing sink [71].

**Figure 2:**
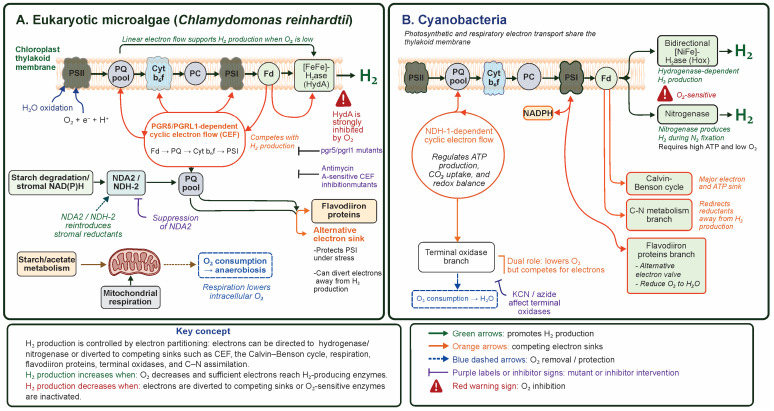
Alternative electron transfer pathways and competing metabolic sinks regulating H_2_ production in microalgae and cyanobacteria. Created with CorelDRAW 2020.

Overall, these findings show that photosynthetic regulation of H_2_ production is more complex than simple suppression of PSII-derived O_2_ evolution. Efficient H_2_ production requires simultaneous control of O_2_ concentration, proton-gradient formation, stromal redox state, carbon fixation, starch metabolism, respiration, cyclic electron flow, and alternative routes of plastoquinone reduction. Therefore, future strategies should aim not at complete inhibition of photosynthesis, but at rational redirection of electron flow away from competing sinks and toward H_2_ase while preserving sufficient photosynthetic activity to provide reductant (Figure 2).

### 2.5. Comparative Analysis and Mechanistic Insights

It is important to distinguish between H_2_ production pathways in prokaryotic cyanobacteria and eukaryotic microalgae. In cyanobacteria, H_2_ evolution is primarily mediated by N_2_ase and bidirectional H_2_ase, which are linked to respiratory and photosynthetic electron transport processes. In contrast, microalgae rely mainly on [FeFe]-H_2_ase, which directly accepts electrons from photosystem I via ferredoxin [72,73]. PSII inhibitors are used to test how PSII-derived O_2_ evolution and PSII-derived electron supply influence H_2_ production. Their effects are inherently dual. By suppressing water oxidation, they can reduce O_2_ accumulation and protect O_2_-sensitive H_2_ases. At the same time, they can decrease the flow of electrons from water to the PQ pool, PSI, ferredoxin, and H_2_ase. Therefore, PSII inhibitors should not be interpreted simply as anaerobiosis-inducing agents, but as tools for separating the effects of O_2_ suppression from the effects of electron-supply limitation.

All three classes of inhibitors—PSII inhibitors (atrazine and DCMU), intersystem inhibitors (DBMIB), and proton uncouplers (CCCP)—affect H_2_ production through different mechanisms and should not be considered functionally equivalent in promoting H_2_ evolution. While all can reduce O_2_ evolution to varying degrees, their effects on electron flow toward H_2_ase differ substantially. Atrazine, like DCMU, inhibits electron transfer at the PSII Q_B acceptor side, reducing O_2_ evolution while also restricting PSII-derived electron supply to H_2_ase [74,75]. As a result, H_2_ evolution under PSII inhibition is highly context-dependent and may rely on residual PSII activity or alternative electron sources. Therefore, atrazine is more appropriately considered a mechanistic comparator rather than a consistently effective inducer of H_2_ production. In contrast, DBMIB blocks the cytochrome b_6_f–PSI leg. PSII can still split water, but its electron flow cannot reach PSI. This leads to an over-reduced plastoquinone pool, which gradually downregulates PSII and O_2_ evolution [76,77]. The effect is a more gradual onset of anoxia, often sustaining low but continuous H_2_ evolution, as observed in *C. reinhardtii* cultures treated with DBMIB. Importantly, DBMIB may either suppress or prolong H_2_ production depending on concentration, timing, and physiological state. In contrast, CCCP dissipates the proton gradient rather than targeting a specific electron transport step, thereby indirectly suppressing PSII activity through elimination of the ΔpH component of the proton motive force. By uncoupling electron transport from ATP synthesis, CCCP rapidly alters intracellular redox balance and accelerates the establishment of anaerobic conditions favorable for H_2_ase activity [50,78]. Accordingly, CCCP-treated algae typically exhibit rapid and strong short-term stimulation of H_2_ production, rather than prolonged H_2_ evolution. These differences are reflected in practical outcomes (Table 2). Among the tested inhibitors, CCCP has been reported to exert the most significant effect on increasing H_2_ yield, whereas DBMIB and DCMU tend to prolong the duration of H_2_ production. For example, the addition of DCMU has been shown to approximately halve H_2_ output, while DBMIB can strongly suppress H_2_ production, underscoring the sensitivity of PSII-dependent electron flow to different types of inhibition [50,78]. However, this apparent suppression depends strongly on experimental conditions, and in many cases, DBMIB supports sustained H_2_ production rather than completely inhibiting it. Notably, the effects of these inhibitors are often contradictory and strongly depend on experimental conditions. While DCMU and DBMIB may prolong H_2_ production, they can also inhibit H_2_ evolution under certain conditions. Therefore, their impact should be interpreted as context-dependent rather than universally beneficial. Recent studies emphasize that efficient H_2_ production depends on maintaining a dynamic balance between electron flow and O_2_ consumption, rather than complete inhibition of photosynthesis. In this context, modulation of proton gradients and intracellular redox conditions has been shown to prolong H_2_ase activity even when PSII function is only partially suppressed [66,71]. It should be noted that the effects of photosynthesis inhibitors are strongly concentration-dependent. At low concentrations, compounds such as DCMU and CCCP may promote H_2_ production by partially suppressing O_2_ evolution while maintaining sufficient electron flow. In contrast, at higher concentrations, these inhibitors can fully block electron transport or severely disrupt cellular metabolism, resulting in inhibition of H_2_ production. For example, DCMU is typically effective at low micromolar concentrations (∼0.5–5 μM), whereas CCCP often stimulates H_2_ production in the range of 5–20 μM but becomes inhibitory at higher levels. Therefore, identifying optimal concentration ranges is critical for maximizing H_2_ production efficiency [60,79,80].

## 3. Photosynthesis Inhibitors of Cyanobacteria and Their Targets

A variety of metabolic and cultivation-based strategies have been explored to enhance H_2_ production in cyanobacteria. These include optimization of medium composition, such as varying the concentrations of NaNO_3_, MgSO_4_, NaCl, Fe^3+^, and Ni^2+^ [83], as well as adjustment of carbon sources, pH, buffer systems (TES, HEPES, and TRIS), temperature, and light regime [84]. Among chemical approaches, photosynthetic and respiratory inhibitors have received attention because they can reveal how O_2_ evolution, respiration, terminal oxidases, and competing electron sinks regulate H_2_ production. In selected cases, these compounds may increase H_2_ yield, but their effects are strongly dependent on dose, organism, metabolic state, and whether H_2_ production is mediated by N_2_ase or H_2_ase.

The importance of inhibitors in cyanobacterial bioH_2_ research is closely linked to one of the major physiological constraints of this process: the extreme O_2_ sensitivity of the enzymes responsible for H_2_ formation, especially bidirectional H_2_ase and N_2_ase. Therefore, compounds that suppress O_2_ evolution, redirect electrons away from competing pathways, or modify cellular energy metabolism can strongly influence H_2_ yield. In cyanobacteria, however, the interpretation of inhibitor effects is often complicated because photosynthetic and respiratory electron transport are tightly interconnected and share the thylakoid membrane. Among the compounds most frequently used in this context are DCMU, CCCP, DBMIB, and KCN.

### 3.1. Photosystem II Inhibitors in Cyanobacteria: DCMU, Atrazine, and Simazine

The best-characterized class of inhibitors used in cyanobacterial H_2_ studies targets PSII, particularly the acceptor side of the reaction center. DCMU, together with the triazine herbicides atrazine and simazine, inhibits electron transfer by competing with plastoquinone for the Q_B_-binding niche of the D1 protein. This blocks electron transfer from Q_A_ to Q_B_, interrupts linear electron flow from water to the plastoquinone pool, and consequently suppresses PSII-dependent O_2_ evolution. Structural and biochemical analyses have shown that the Q_B_ site is highly conserved in oxygenic phototrophs, which explains the broad effectiveness of these inhibitors in cyanobacteria, algae, and plants [85].

Atrazine and simazine act at the same general target as DCMU, although they have been less extensively studied in cyanobacterial H_2_ production systems. Nevertheless, available evidence indicates that both compounds can enhance H_2_ evolution under suitable conditions. In *Aphanothece halophytica*, both atrazine and simazine stimulated photobiological H_2_ production, while simazine produced the strongest overall response. In particular, 25 μM simazine markedly reduced photosynthetic O_2_ evolution, promoted dark respiration, lowered intracellular O_2_ tension, and increased bidirectional H_2_ase activity, resulting in a maximum H_2_ production rate of 58.88 μmol H_2_ g^−1^ dry weight h^−1^ and several-fold higher H_2_ accumulation. These findings suggest that, although DCMU, atrazine, and simazine all function as PSII inhibitors, simazine may be especially useful when prolonged suppression of O_2_ production is required.

Among PSII inhibitors, DCMU remains the most widely used mechanistic probe. By blocking electron transfer between Q_A_ and Q_B_, DCMU suppresses water oxidation-derived O_2_ formation and creates a more favorable intracellular environment for O_2_-sensitive H_2_-producing enzymes [26]. DCMU has been shown to inhibit PSII activity in several cyanobacteria, including *Aphanocapsa* sp. 6308, *Nostoc* sp., and *Lyngbya* sp., as well as in green algae. In addition to its direct effect on PSII, DCMU may also influence cyclic phosphorylation, chlorophyll biosynthesis, fatty acid synthesis, and respiratory metabolism, indicating that its physiological effects are broader than simple PSII inhibition alone [60].

Previous studies have demonstrated the impact of DCMU on H_2_ production in cyanobacterial strains, including *Sodalinema gerasimenkoae* IPPAS B-353, *Dolichospermum* sp. IPPAS B-1213, *Cyanobacterium* sp. IPPAS B-1200, and *Synechocystis* sp. PCC 6803 GT 926) [86]. In this study, they investigated the effect of DCMU on H_2_ production rates under artificial lighting conditions.

From the perspective of H_2_ production, DCMU is valuable because it helps distinguish whether H_2_ evolution is mainly limited by O_2_ release or by electron supply. In *Aphanothece halophytica*, DCMU increased light-driven H_2_ production by approximately three- to fivefold, an effect associated with reduced chlorophyll fluorescence and lower O_2_ levels that favored bidirectional H_2_ase activity. Similar stimulation has been reported in *Anabaena siamensis* TISTR 8012, where 50 μM DCMU increased H_2_ production about threefold, and in *Cyanothece* sp. Miami BG 043511, where photo-H_2_ production increased 2.35-fold after suppression of PSII-dependent O_2_ evolution [60]. Thus, DCMU can enhance cyanobacterial H_2_ production in some systems, but its broader value is as an informative probe for distinguishing whether H_2_ evolution is limited primarily by O_2_ accumulation, PSII-derived electron supply, or competing electron sinks.

### 3.2. Inhibitors of Intersystem Electron Transport in Cyanobacteria: DBMIB

DBMIB is classically used as an inhibitor of electron transfer between PSII and PSI. Its principal target is the PQH_2_ oxidation site (Q_o_ site) of the cytochrome b_6_f complex, where it blocks oxidation of reduced plastoquinone and thereby prevents efficient electron transfer from the plastoquinone pool to PSI. In cyanobacterial H_2_ studies, DBMIB is particularly informative because it can reveal whether the reductant must pass through cytochrome b_6_f and PSI before reaching the H_2_-producing machinery.

Evidence from cyanobacterial systems indicates that DBMIB can strongly suppress light-driven H_2_ formation. In *Cyanothece*, DBMIB blocked electron transfer from reduced plastoquinone to PSI and abolished photo-H_2_ production, suggesting that the light-dependent pathway was PSI-dependent and predominantly N_2_ase-mediated. Similarly, in *Synechocystis* sp. PCC 6803, DBMIB suppressed light-driven H_2_, O_2_, and CO_2_ exchange, demonstrating that reoxidation of the plastoquinone pool by cytochrome b_6_f is essential for these processes [87]. These observations make DBMIB a useful tool for probing the role of intersystem electron transport in cyanobacterial H_2_ metabolism.

However, the mechanism of action of DBMIB should be interpreted with caution due to its additional side effects. In addition to inhibiting PQH_2_ oxidation at cytochrome b_6_f, it can also quench PSII chlorophyll excited states and, under some conditions, function as an alternative PSII electron acceptor. Because these side effects occur within the same concentration range commonly used for cytochrome b_6_f inhibition, DBMIB is not a perfectly specific inhibitor. Consequently, it is best regarded as a powerful but mechanistically complex probe of the electron transport segment linking PSII and PSI [88].

### 3.3. Proton Gradient Uncouplers in Cyanobacteria: CCCP

Unlike DCMU or DBMIB, CCCP is more accurately described as a protonophore uncoupler than as a site-specific photosynthetic inhibitor. Its primary action is to dissipate the transmembrane proton gradient, thereby uncoupling electron transport from ATP synthesis and collapsing the proton motive force required for photophosphorylation and oxidative phosphorylation [89]. Because of this broader mode of action, CCCP can indirectly reduce O_2_ accumulation and promote H_2_ production, but its physiological effects extend beyond the photosynthetic apparatus itself.

CCCP has been reported to inhibit PSII photochemical activity in several cyanobacteria and green algae, leading to decreased O_2_ evolution. At the same time, by dissipating the proton gradient, CCCP interferes with ATP synthesis and can redirect electrons and protons toward bidirectional H_2_ase activity. Enhanced H_2_ production in the presence of CCCP has been observed in cyanobacteria such as *Oscillatoria chalybea* and *Synechocystis* sp. PCC 6803, as well as in several green algae. In some cases, CCCP also increases dark respiration, further contributing to O_2_ depletion [60].

This broader physiological action is reflected in experimental outcomes. In *Aphanothece halophytica*, CCCP increased light-driven H_2_ production by approximately three- to fivefold, similar to DCMU. However, unlike DCMU, CCCP also stimulated dark fermentative H_2_ production, which is consistent with its role as an uncoupler of oxidative phosphorylation. The highest response was observed at 0.5 μM CCCP and was associated with lower O_2_ levels and enhanced bidirectional H_2_ase activity [60]. Therefore, CCCP is useful for testing whether H_2_ production is constrained by proton-coupled energy conservation or by O_2_ sensitivity, although its pleiotropic effects make it less suitable than DCMU for precise pathway assignment.

### 3.4. Respiratory Inhibitors in Cyanobacteria: KCN and Related Compounds

The effect of KCN on H_2_ production should be interpreted through the balance between respiratory electron sinks and respiratory O_2_ removal [90]. KCN inhibits cyanide-sensitive terminal oxidases and thereby reduces the ability of respiration to consume electrons and O_2_ [91]. In cyanobacteria, this is especially important because photosynthetic and respiratory electron transport chains share thylakoid redox carriers. Inhibition of terminal oxidases can redirect reductant away from respiration and toward H_2_-producing enzymes, but it can also reduce respiratory O_2_ scavenging and thereby increase inhibition of oxygen-sensitive hydrogenases or nitrogenases. Consequently, KCN can either stimulate or suppress H_2_ production depending on strain, inhibitor concentration, light conditions, respiratory activity, and whether H_2_ formation is mainly hydrogenase- or nitrogenase-dependent [92,93].

Respiratory inhibitors occupy a distinct place in cyanobacterial H_2_ research because respiration in cyanobacteria is not spatially separated from photosynthesis as it is in plants and mitochondria. Instead, photosynthetic and respiratory electron transport coexist in the thylakoid membrane and share several electron carriers and redox components. Cyanobacteria possess multiple terminal respiratory oxidases, including aa_3_-type cytochrome c oxidase, cytochrome bd quinol oxidase, and alternative respiratory terminal oxidases. In *Synechocystis* sp. PCC 6803, KCN inhibits all three terminal oxidases, although with different apparent sensitivities, thereby strongly altering electron sink distribution and intracellular redox balance [60].

KCN is the most frequently discussed respiratory inhibitor in cyanobacterial H_2_ studies. Its stimulatory effect is generally explained by inhibition of terminal oxidases, which reduces electron consumption by respiration and redirects reducing equivalents toward H_2_ase- or N_2_ase-dependent H_2_ production. However, in cyanobacteria, the mechanism of KCN action is more complex than simple inhibition of a single respiratory pathway. Cyanobacteria possess multiple terminal oxidases, including cytochrome aa_3_-type oxidase, cytochrome bd quinol oxidase, and alternative oxidases, which exhibit different sensitivities to KCN. As a result, the extent and specificity of inhibition depend strongly on the applied concentration. Reported KCN concentrations vary widely across studies, and its physiological effects are highly dose-dependent. In addition to inhibiting terminal oxidases, KCN may exert non-specific effects by interacting with other metalloproteins and components of cellular metabolism, thereby altering intracellular redox balance beyond respiratory electron transport [25,94]. Therefore, caution should be exercised when interpreting KCN-induced stimulation of H_2_ production, as the observed effects may reflect a combination of specific respiratory inhibition and broader metabolic perturbations. In *Anabaena siamensis* TISTR 8012, 20 mM KCN increased H_2_ production approximately threefold and was associated with increased Hox-H_2_ase activity, upregulation of *nifD*, and downregulation of *hupL* [95]. In another study on non-heterocystous cyanobacteria, 500 μM KCN increased H_2_ production in *Synechocystis* sp. PSU 1262 by 14.2-fold under optimized light-dark conditions [93]. These results support the view that respiratory terminal oxidases act as important competing electron sinks during H_2_ metabolism, although their relative contribution depends on physiological conditions and inhibitor dosage.

Other respiratory inhibitors have also been used, although generally in a more exploratory manner. Sodium azide has been applied to inhibit azide-sensitive terminal oxidase activity, while rotenone has been used as a probe of NDH-1-like respiratory dehydrogenases, although its specificity in cyanobacteria is not always complete [93]. In *Aphanothece halophytica*, sodium azide enhanced photobiological H_2_ production, whereas rotenone stimulated dark fermentative H_2_ production, suggesting that different respiratory branches compete with H_2_-forming pathways under different physiological conditions (Table 3).

Recent mechanistic advances have highlighted the complexity of H_2_ production in cyanobacteria beyond classical inhibitor-based approaches [72,96]. In particular, alternative electron sinks such as flavodiiron proteins (FDPs), NDH-1 complexes, and terminal oxidases play critical roles in regulating intracellular redox balance and O_2_ consumption. These pathways compete with H_2_ase and N_2_ase for reducing equivalents, thereby limiting H_2_ production under certain conditions. Recent studies have shown that targeted modulation of these competing pathways, including suppression of alternative electron sinks and optimization of cyclic electron flow around PSI, can significantly enhance electron partitioning toward H_2_-evolving enzymes [97,98]. These findings emphasize that cyanobacteria represent highly flexible and engineerable platforms for sustainable H_2_ production.

**Table 3:** Effects of metabolic inhibitors on H_2_ production in cyanobacterial strains.

Strain	Inhibitor Concentration	H_2_ Production Condition	H_2_ Maximum Rate	Ref.
DCMU				
*Anabaena siamensis* TISTR 8012	50 μM	DL-glyceraldehyde under light condition for 24 h	22 μmol H_2_/mg *Chl* a/h	[93]
*Cyanobacterium* sp. IPPAS B-1200	20 μM	Under light anaerobic conditions on the first day	Detectable H_2_	[86]
*Dolichospermum* sp. IPPAS B-1213	20 μM	Under light anaerobic conditions, after 24 h	4.24 mmol H_2_/mg *Chl* a/h	[86]
*Sodalinema gerasimenkoae* IPPAS B-353	10 μM	Under dark anaerobic conditions, after 24 h	0.45 μmol H_2_/mg *Chl* a/h	[86]
*Synechocystis* sp. PCC 6803 GT-L	20 μM	Under dark anaerobic conditions, after 24 h.	0.72 μmol H_2_/mg *Chl* a/h	[86]
*Synechococcus elongatus* sp. 7942 expressing HydA	5 μM	Headspace: 2.5% CO_2_ in N_2_, light condition for 96 h	250–260 μmol H_2_/mg *Chl* a/h	[99]
*Anabaena* sp. PCC 7120	1.0 μM	Anaerobic continuous light	77 μmol H_2_/mg *Chl* a/h	[100]
*Synechocystis* sp. PSU 1262	20 μM	Dark fermentative for 48 h	710 nmol H_2_/mg *Chl* a/h	[25]
CCCP				
*Aphanothece halophytica*	0.5 μM	Dark fermentative for 2 h	39.50 *μ*mol H_2_/g dry weight/h	[60]
*Synechocystis* sp. PSU 1262	1 μM	Dark fermentative for 48 h	158.3 nmol H_2_/mg *Chl* a/h	[25]
DBMIB				
*Synechococcus elongatus* sp. 7942 expressing HydA	20 μM	Headspace: 2.5% CO_2_ in N_2_, light condition for 96 h	H_2_ production was strongly reduced	[99]
*Synechocystis* sp. PSU 1262	20 μM	Dark fermentative for 48 h	96.8 nmol H_2_/mg *Chl* a/h	[25]
*Synechocystis* sp. PCC 6803 GT	110 μM	light anaerobic incubation: 100–110 μmol photons m^−2^ s^−1^	1.72 μmol H_2_/mg *Chl* a/h	[88]
KCN				
*Anabaena siamensis* TISTR 8012	20 mM	DL-glyceraldehyde under light condition for 24 h	22 μmol H_2_/mg *Chl* a/h	[93]
*Synechocystis* sp. PSU 1262		500 μM	860 nmol H_2_/mg *Chl* a/h	[25]
Simazine				
*Aphanothece halophytica*	25 μM	Under dark anaerobic conditions for 24 h	356.21 ± 6.04 μmol H_2_/g dry weight/h	[101]
Tungstate				
*Synechocystis* sp. PCC *6803* ΔctaIΔcydΔctaII	4.8 μM	Under dark anaerobic conditions for 24 h	200 nmol H_2_/mg *Chl* a/h	[102]

## 4. Future Perspectives

Future progress in this field will likely depend not on the discovery of increasingly potent photosynthetic blockers, but rather on the development of precise, reversible, and context-dependent modulators of photosynthetic electron transport. Classical inhibitors such as DCMU, DBMIB, and CCCP have been invaluable for dissecting the relationship between O_2_ evolution, redox balance, and H_2_ production. However, their usefulness as translational tools remain limited because they often induce broad physiological perturbations, suppress biomass productivity, and generate secondary effects that complicate mechanistic interpretation. For this reason, the next generation of studies should move beyond the concept of complete pathway inhibition and focus instead on fine-tuning electron partitioning toward H_2_-evolving enzymes while preserving sufficient photosynthetic activity to sustain reductant supply.

A particularly promising direction is the search for novel small molecules targeting regulatory bottlenecks rather than major catalytic nodes of photosynthesis. Instead of fully blocking PSII, future compounds should ideally allow partial suppression of O_2_ evolution while maintaining residual electron flow or stimulating alternative electron donation pathways. This concept is especially relevant for H_2_ photoproduction, where maximal inhibition of PSII often reduces intracellular O_2_ but simultaneously deprives H_2_ase of electrons. Therefore, compounds that subtly modulate the plastoquinone pool, cyclic electron flow around PSI, chlororespiratory pathways, or the proton motive force may be more effective than conventional PSII herbicides. In cyanobacteria, similar attention should be given to molecules affecting terminal oxidases, flavodiiron proteins, NDH-dependent electron transfer, and other competing sinks that influence intracellular redox poise and O_2_ consumption. Examples of such modulators include respiratory inhibitors such as KCN and sodium azide, which target terminal oxidases, as well as redox-active compounds such as paraquat and DBMIB that affect electron transport and intracellular redox balance.

Another important future direction involves the identification of species-specific and condition-specific modulators. The effects of photosynthesis inhibitors are clearly not universal; they depend strongly on organism type, physiological state, light regime, nutrient availability, and the relative contribution of PSII-dependent versus PSII-independent H_2_ production pathways. This means that screening efforts should no longer rely solely on compounds borrowed from plant herbicide research. Instead, there is a need for dedicated chemical libraries designed specifically for photobiological H_2_ systems, combined with high-throughput phenotyping, chlorophyll fluorescence profiling, metabolomics, and redox-state analysis. Such an approach could reveal compounds that do not simply inhibit photosynthesis but selectively reprogram electron allocation under H_2_-producing conditions.

In parallel, future research should integrate chemical modulation with bioengineering and materials-based strategies. Recent advances suggest that H_2_ production can be improved not only by altering electron transport chemically, but also by creating localized hypoxic microenvironments, immobilizing cells, engineering alternative electron transfer routes, or redesigning photosynthetic control networks through synthetic biology. In this context, inhibitors should be viewed not as standalone solutions, but as components of integrated systems in which low-dose chemical modulation is combined with cell immobilization, conductive materials, metabolic engineering, or inducible genetic control. Such hybrid approaches may provide a more stable balance between O_2_ suppression and electron availability than any single inhibitor alone [54].

Future progress in this field will increasingly depend on integrating chemical, metabolic, and engineering-based strategies [103]. In particular, metabolic flux analysis and redox balancing approaches can be used to optimize the distribution of reducing equivalents toward H_2_-producing pathways. Synthetic biology tools, including targeted gene editing and pathway rewiring, offer opportunities to suppress competing electron sinks and enhance H_2_ase or N_2_ase activity [104]. In addition, cell immobilization and the development of engineered microenvironments can improve stability, prolong H_2_ production, and enable more efficient light and electron utilization [105,106]. Finally, multi-omics approaches, including transcriptomics, proteomics, and metabolomics, are expected to play a key role in identifying regulatory bottlenecks and guiding rational design of high-performance H_2_-producing systems [107,108].

Finally, future work should place greater emphasis on sustainability, reversibility, and scalability. Many classical inhibitors are toxic, poorly selective, or unsuitable for large-scale use. The long-term goal should therefore be the development of environmentally compatible modulators that act transiently, are active at low concentrations, and can be precisely controlled in time and space. In this sense, the field is gradually shifting from the study of “photosynthesis inhibitors” in the classical sense toward the broader concept of photosynthetic flow control. Advancing this transition will be essential for transforming inhibitor-based experiments from mechanistic laboratory tools into realistic strategies for efficient biological H_2_ production in microalgae and cyanobacteria. In addition, the development of mutant and genetically engineered strains represents a promising direction to enhance photobiological H_2_ production [109,110]. Targeted modifications of competing electron sinks, photosynthetic pathways, and redox regulation can improve electron allocation toward H_2_-producing enzymes, supporting more efficient and stable H_2_ evolution [111,112].

## 5. Conclusions

Photosynthesis inhibitors are valuable tools for understanding and controlling H_2_ production in microalgae and cyanobacteria. Their impact depends on several factors, including where they act in the photosynthetic system, how much is used, the type of organism, and the growth conditions. Inhibitors such as DCMU and atrazine mainly reduce O_2_ production by blocking PSII, which can be helpful because O_2_ often interferes with H_2_-producing enzymes. However, if the inhibition is too strong, it can also reduce the flow of electrons needed for H_2_ generation. On the other hand, compounds like DBMIB and CCCP may enhance H_2_ production in certain conditions by affecting electron transport, proton balance, and the overall redox state of the cell. In cyanobacteria, respiratory inhibitors such as KCN also show how strongly H_2_ yield is influenced by other pathways that compete for electrons. Microalgae and cyanobacteria share common principles of H_2_ production but differ in their electron transport organization. Microalgae rely mainly on [FeFe]-H_2_ase, whereas cyanobacteria possess more flexible systems involving N_2_ase and respiratory pathways, making them more suitable for engineered H_2_ production. Successful photobiological H_2_ production depends on finding the right balance: reducing O_2_ buildup while still maintaining enough electron flow for H_2_-producing enzymes to function efficiently. Future studies should therefore focus on carefully optimizing inhibitor types and concentrations, while combining these approaches with metabolic engineering and other bioengineering strategies to achieve more stable and efficient H_2_ production.

## Figures and Tables

**Table 1:** Photosynthetic inhibitor targets and their mechanistic effects on H_2_ production.

Inhibitor	Main Target	Immediate Biochemical Effect	Redox Consequence	Expected Effect on H_2_ Production
DCMU	PSII acceptor side; D1/QB region	Blocks from Q_A_ to Q_B_ electron transfer; closes PSII reaction centers; suppresses PSII-dependent linear electron flow and O_2_ evolution	Restricts electron entry into PQ/PQH_2_ pool; limits PSII-derived electrons to PSI, Fd, and hydrogenase	Usually decreases direct PSII-dependent H_2_; may transiently help only when O_2_ suppression outweighs loss of electron supply and alternative donors remain active
Atrazine	PSII acceptor side; D1/QB region; triazine-class inhibitor	Blocks Q_A_ → Q_B_ electron transfer and decreases PSII quantum efficiency and ETR	Produces PSII acceptor-side reduction and altered fluorescence/ETR responses in a dose- and species-dependent manner	Best used as a mechanistic comparator for PSII acceptor-side inhibition; not a generally reliable H_2_-enhancing strategy
DBMIB	Primary: cytochrome b_6_f Qo site; additional PSII/quinone-mediated effects reported	Blocks PQH_2_ oxidation and restricts electron transfer from PQ pool to PC/PSI; may also perturb PSII fluorescence and charge recombination	PQ pool becomes more reduced; PSI, Fd, and hydrogenase can become electron-limited; interpretation complicated by DBMIB side effects	Strong inhibition usually suppresses H_2_ by limiting PSI–Fd electron delivery; low-dose partial inhibition may prolong suboxic H_2_ only in a narrow context-dependent window
CCCP	Proton motive force across thylakoid membrane	Collapses ΔpH/pmf; uncouples electron transport from ATP synthesis	Alters ATP/NADPH balance, photosynthetic control, O_2_ evolution, and stromal electron allocation	Can stimulate short-term H_2_ under some conditions, but effect is pleiotropic and not site-specific
KCN	Cyanide-sensitive terminal oxidases; respiratory electron sinks	Inhibits respiratory terminal oxidation and O_2_ consumption	Reduces competing respiratory electron sinks but may also reduce O_2_ scavenging	May stimulate H_2_ when competing respiratory sinks dominate, but may suppress H_2_ if respiratory O_2_ scavenging becomes limiting

**Table 2:** Comparative effects of photosynthesis inhibitors and related photosynthetic modulation strategies on H_2_ production in microalgae.

Inhibitor	Biological Model	Molecular Target	Experimental Setup	Observed H_2_ Response	Quantitative Performance	Ref.
DCMU	*C. reinhardtii*	PSII acceptor side (from Q_A_ to Q_B_)	High H_2_-producing strains under illumination	Decrease in H_2_ production	~50% reduction in H_2_ evolution rate	[50,78]
DCMU	*C. pyrenoidosa*	PSII	Dose-dependent addition under non-stress conditions	Temporary increase or delay effect reported	Variable (condition-dependent)	[81,82]
Atrazine	*P. kessleri*	PSII Q_B_ site	Sub-micromolar exposure	Not directly linked to H_2_ production	Strong decline in PSII efficiency (Fv/Fm, ΦPSII)	[44]
Atrazine	*Chlorella* sp.	PSII Q_B_ site	Dose–response exposure	Reduced photosynthetic activity	Decline in ETR	[45]
DBMIB	*C. reinhardtii*	Cytochrome b_6_f (Qo site)	3.5 μM DBMIB	Sustained H_2_ production	Up to 30 days production; peak ~23 mL H_2_ L^−1^ day^−1^	[54]
DBMIB + immobilization	*C. reinhardtii* (alginate beads)	Cytochrome b_6_f	Immobilized cells + DBMIB	Increased H_2_ yield	~200 μmol H_2_ mg^−1^ Chl over 3 weeks (~2× higher)	[55]
CCCP	*C. reinhardtii*	Proton gradient (ΔpH)	15 μM CCCP treatment	Strong increase in H_2_ production	~13-fold increase; sustained ≥12 h	[59]
PSII modulation (partial)	*C. reinhardtii*	PSII (regulated activity)	Sulfur deprivation/controlled PSII downregulation	Sustained H_2_ production	Long-term stable production (no exact single value)	[34,35]

## Data Availability

The data that support the findings of this study are available from the corresponding authors upon reasonable request.

## Abbreviations

ATP	Adenosine triphosphate
ATPase	ATP synthase
CCCP	Carbonyl cyanide *m*-chlorophenyl hydrazone
Chl	Chlorophyll
Chl *a*	Chlorophyll *a*
CO_2_	Carbon dioxide
Cyd	Cytochrome *bd*-type oxidase
Cyt *b_6_f*	Cytochrome *b_6_f* complex
Cyt ox	Cytochrome oxidase
D1	D1 protein of photosystem II
DBMIB	2,5-dibromo-3-methyl-6-isopropyl-*p*-benzoquinone
DCMU	3-(3,4-dichlorophenyl)-1,1-dimethylurea
DMSO	Dimethyl sulfoxide
ETR	Electron transport rate
Fd	Ferredoxin
FNR	Ferredoxin–NADP^+^ reductase
Fv/Fm	Maximum quantum efficiency of photosystem II
H_2_	Hydrogen
H_2_ase	Hydrogenase
HEPES	Buffer used in this study
HydA	[FeFe]-hydrogenase
HydA1	Hydrogenase isoform 1
HydA2	Hydrogenase isoform 2
KCN	Potassium cyanide
N_2_	Nitrogen
N_2_ase	Nitrogenase
NAD(P)H	Reduced nicotinamide adenine dinucleotide phosphate/nicotinamide adenine dinucleotide
NDH	NAD(P)H dehydrogenase
NDH-1	NAD(P)H dehydrogenase-like complex 1
O_2_	Oxygen
PC	Plastocyanin
pmf	Proton motive force
PPy	Polypyrrole
PQ	Plastoquinone
PQH_2_	Plastoquinol
PSI	Photosystem I
PSII	Photosystem II
Q_A	Primary quinone electron acceptor of photosystem II
Q_B	Secondary quinone electron acceptor of photosystem II
Q_o	Quinol oxidation site of the cytochrome *b_6_f* complex
ROS	Reactive oxygen species
TES	Buffer used in this study
TRIS	Buffer used in this study
Y(II)	Effective quantum yield of photosystem II
ΔpH	Trans-thylakoid proton gradient
ΦPSII	Effective quantum yield of photosystem II
